# Written versus verbal consent: a qualitative study of stakeholder views of consent procedures used at the time of recruitment into a peripartum trial conducted in an emergency setting

**DOI:** 10.1186/s12910-017-0196-7

**Published:** 2017-05-24

**Authors:** J. Lawton, N. Hallowell, C. Snowdon, J. E. Norman, K. Carruthers, F. C. Denison

**Affiliations:** 1 Centre for Population Health Sciences, University of Edinburgh, Edinburgh, UK; 2 Ethox Centre, Nuffield Department of Population Health, University of Oxford, Oxford, UK; 3 Department of Medical Statistics, London School of Hygiene and Tropical Medicine, London, UK; 4 MRC Centre for Reproductive Health, University of Edinburgh, Edinburgh, UK

**Keywords:** Consent, Qualitative, Peripartum, Emergency setting, Ethics

## Abstract

**Background:**

Obtaining prospective written consent from women to participate in trials when they are experiencing an obstetric emergency is challenging. Alternative consent pathways, such as gaining verbal consent at enrolment followed, later, by obtaining written consent, have been advocated by some clinicians and bioethicists but have received little empirical attention. We explored women’s and staff views about the consent procedures used during the internal pilot of a trial (GOT-IT), where the protocol permitted staff to gain verbal consent at recruitment.

**Methods:**

Interviews with staff (*n* = 27) and participating women (*n* = 22). Data were analysed thematically and interviews were cross-compared to identify differences and similarities in participants’ views about the consent procedures used.

**Results:**

Women and some staff highlighted benefits to obtaining verbal consent at trial enrolment, including expediting recruitment and reducing the burden on those left exhausted by their births. However, most staff with direct responsibility for taking consent expressed extreme reluctance to proceed with enrolment until they had obtained written consent, despite being comfortable using verbal procedures in their clinical practice. To account for this resistance, staff drew a strong distinction between research and clinical care and suggested that a higher level of consent was needed when recruiting into trials. In doing so, staff emphasised the need to engage women in reflexive decision-making and highlighted the role that completing the consent form could play in enabling and evidencing this process. While most staff cited their ethical responsibilities to women, they also voiced concerns that the absence of a signed consent form at recruitment could expose them to greater risk of litigation were an individual to experience a complication during the trial. Inexperience of recruiting into peripartum trials and limited availability of staff trained to take consent also reinforced preferences for obtaining written consent at recruitment.

**Conclusions:**

While alternative consent pathways have an important role to play in advancing emergency medicine research, and may be appreciated by potential recruits, they may give rise to unintended ethical and logistical challenges for staff. Staff would benefit from training and support to increase their confidence and willingness to recruit into trials using alternative consent pathways.

**Trial Registration:**

This qualitative research was undertaken as part of the GOT-IT Trial (trial registration number: ISCRTN 88609453). Date of registration 26/03/2014.

## Background

The challenges of recruiting and gaining consent to participate in clinical trials are well recognised, especially when the research is undertaken in emergency situations or in particular patient groups such as pregnant or recently pregnant women [1–3]. This is because the ability of the person to make an informed decision may be partly or severely compromised [4, 5]. There may also be very limited time in which to undertake recruitment, and/or recruiting staff may feel it is inappropriate to approach family members/proxies due to their being distracted and distressed [6, 7]. To help address these challenges, and enable trial research to be undertaken in emergency situations without delaying treatment, more flexible approaches to gaining consent have been advocated and developed, which balance the need for consent and participant protection against the need for research to be undertaken [8, 9]. At one extreme, this involves use of consent waivers where participants are recruited to trials without consent being taken [5, 8]. However, deferred consent (i.e. enrolling an individual into a trial and seeking consent retrospectively) tends to be a preferred approach, especially in clinical trials where obtaining prospective consent might lead to harmful delays to the initiation of treatment [4, 6, 7]. While ethical concerns have been raised about not informing patients/proxies about trial participation, or only doing so retrospectively, consent waivers and deferred consent procedures have been seen to play an important role in advancing trial research and, hence, the development of treatments used in emergency settings [8, 10]. As well as being the subject of ethical attention and debate [8, 10–13], use of consent waivers and deferred consent procedures has received some empirical attention. For instance, questionnaire and qualitative studies have reported patient/proxy [7, 14–16] and health professional [7, 15, 17, 18] support for use of deferred consent in emergency research, although it has also been found that health professionals may initially be reticent about using what they see as an unorthodox approach [18–20]. Studies which have explored patient, public and professional opinions about use of consent waivers have revealed a much more ambivalent picture, with research regulators being more cautious about their use than patients and health professionals [5, 15].

Alternative scenarios for gaining consent, which potentially a steer the middle ground, have received surprisingly little empirical attention. This includes use of verbal consent at the time of trial enrolment followed, at a later stage, by written consent, an approach which has been advocated by some clinicians and bioethicists [21, 22] as it “balances the pressing need to conduct research in the emergency setting with an ethical approach which strives to inform and consult patients before their participation” [22]. Such an approach has also been endorsed by the Royal College of Obstetricians and Gynaecologists in recent guidance about obtaining consent in peripartum (around the time of birth) research where consent is time critical. These guidelines state that: ‘[in acute circumstances]…provision of antenatal information to women with brief oral consent at the time of the complication is appropriate. Full written consent is then obtained at a later stage’ [23]. This approach to gaining consent is being offered as part of an on-going clinical trial, the Glyceryl Nitrate for Retained Placenta (GOT-IT) trial, which involves recruitment of women at a time of obstetric emergency due to them having a retained placenta. As well as using a summary participant information sheet, the trial protocol and associated ethical approvals permit women to give verbal consent at the time of enrolment with written consent taken as soon as possible after trial participation. If a woman chooses to give verbal consent, she is given the summary information leaflet (which she can read if she wants) and the trial is explained to her. If the trained person is happy that the woman understands what trial participation involves, the recruiter is allowed to take her verbal consent. This is recorded in the woman’s medical records and is then followed up by formal written consent as soon as is possible in the postnatal period. The decision to include this approach was made because it was recognised that a retained placenta is a potentially life-threatening complication with the amount of blood loss increasing the longer a placenta is retained. It was also recognised that, due to the emotional and physical impact of birth, women might find it burdensome to complete and sign the form at the time of recruitment and, hence, might prefer to give initial verbal consent. The trial included an internal pilot, during which exploratory qualitative work was undertaken with women and trial staff to explore their views about the trial’s recruitment and consent procedures. One of the objectives of this work was to provide recommendations for use in future trials involving recruitment and consent of individuals in emergency situations.

In an earlier paper [24], we reported women’s and trial staffs’ views about the timing of the recruitment approach, which took place immediately after diagnosis of a retained placenta, and the content and delivery of trial information. As we described, women appreciated being given access to a summary participant information sheet at the time of recruitment, as they considered the full version too lengthy and time-consuming to read. Many also highlighted the benefits of having staff present to explain the trial, as, due to exhaustion, they had found even the summary participant information sheet too burdensome to read. Relatedly, many women described how they had wanted and appreciated opportunities to expedite the recruitment process. This was because they had been eager to try the trial intervention at the earliest opportunity as they thought this might prevent from them from having to go to theatre and, hence, allow them to be there for their baby. For similar reasons, staff also supported use of summary information sheets and verbal information delivery approaches. Not only were these seen to streamline and expedite the recruitment process in a context where there was limited time in which to recruit and consent, staff also suggested that they had enabled information to be imparted in clear and easy to understand ways [24].

In this paper we consider the culmination of the recruitment and consent pathway: the giving and documentation of consent. Specifically, we will consider why, despite some women and staff highlighting the potential benefits of giving verbal consent at the time of recruitment, most staff who had direct responsibility for gaining consent from women into the trial expressed extreme reluctance to proceed with enrolling women into the trial until written consent had been obtained.

### The research setting: The GOT-IT trial

As described previously [24, 25] the GOT-IT trial is a randomised placebo controlled double blind pragmatic RCT involving women who have a retained placenta recruited from delivery wards in the United Kingdom (UK). Retained placenta is a major cause of postpartum haemorrhage and affects around 2% of vaginal deliveries in the UK. It is diagnosed when the placenta is not delivered within 30 min following active management or 60 min after physiological followed by active management of the third stage of labour after delivery of the baby [26]. The aim of the GOT-IT trial is to determine whether use of Glyceryl trinitrate (GTN) spray, as compared to a placebo, can facilitate delivery of the placenta without having to undertake manual or surgical removal of the placenta in theatre. Once a diagnosis of retained placenta has been made, women are recruited into the trial by a member of the clinical or research team who has received appropriate training and has delegated responsibilities to take consent.

The trial comprises an internal pilot followed by a substantive RCT. The pilot ran from October 2014 to April 2015 and involved eight sites, which were entered in a phased way. The purpose of the internal pilot was to provide evidence and reassurance of the feasibility and effectiveness of all trial processes, including those relating to recruitment and obtaining informed consent. The substantive trial is due to be completed in 2017.

## Methods

### Qualitative study design

The qualitative research explored women’s and staff experiences of, and views about, the recruitment and consent procedures used during the pilot, including the giving/soliciting and documentation of informed consent. Full details of the design have been reported previously [24, 25]. In brief, in-depth interviews, informed by topic guides, were used as the method of data collection, as these afforded the flexibility needed for participants to raise and discuss issues that they perceived as salient, while helping to ensure the discussion stayed relevant to addressing the study objectives. Interviews also afforded privacy, allowing participants to share negative views, if they wished to do so. The study employed an iterative approach that entailed simultaneous data collection and analysis [27].

### Sample and recruitment

Recruitment to the qualitative research was undertaken in all eight centres involved in the pilot between November 2014 and April 2015. Women were approached within 2-3 days of having taken part in the trial and were either given a recruitment pack while in hospital, or a pack was posted out if they had already been discharged. Staff were given or sent recruitment packs. The study information sheets for both women and staff made it clear that the interviews were being conducted by independent researcher (NH), who was not a member of the trial team. Individuals were also reassured that all information disclosed would be treated in confidence, with only anonymised findings being fed back to the trial team.

Women were purposively sampled so there was diversity in the final sample in terms of age, education/occupation, parity and ethnicity (see Table 1). Staff were selected for interviews if they had been involved in trial delivery, recruitment or gaining consent from women. Across the centres, these staff comprised research midwives and obstetricians and midwives who were working clinically on the delivery suite. All participants gave their written informed consent to take part in an interview. Recruitment into the qualitative study was necessarily constrained by the relatively short duration of the trial’s pilot phase and the staggered entry of the sites into the pilot; this also limited the number individuals who could be approached to take part into the qualitative study. However, despite these restrictions, it was possible for us to continue to recruit until we got good representation of participants (women and staff) from across the pilot sites and had collected sufficient data to allow a diversity of perspectives and views to be captured and explored in-depth.

**Table 1:** Participant Characteristics – women

	N	%
Age (years)^a^	30.1	18–40
Ethnic Group		
White British	17	77
South East Asian	2	9
Other	3	14
Highest education level		
School	7	32
Further education	2	9
Degree	8	36
Higher degree	5	23
Previous birthing experience		
Primigravidas	9	41
Previous retained placenta	5	39

^a^Mean: Range

### Data collection and analysis

The topic guides for the interviews were developed in the light of literature reviews, inputs from staff and lay advisors, and revised in light of emerging findings. Key areas explored included: women’s experiences of birth and of being approached to take part in the trial; women’s views about consent procedures used and how these might be improved; staff experiences of delivering trials prior to GOT-IT; staff views about the consent procedures used in GOT-IT; and, reasons for using written and/or verbal approaches during the pilot phase.

All interviews were digitally recorded and transcribed in full. Data were analysed thematically by JL and NH using the method of constant comparison [28]. This approach entailed individual interviews being read through repeatedly before being cross-compared to identify issues and experiences (i.e. themes) which cut across different accounts. A comparative analysis of women’s and staff accounts was also undertaken to identify differences and similarities in their views about the consent procedures used. JL and NH undertook independent analyses before meeting to discuss their interpretation of the data, resolve any differences in opinion and to reach agreement on key findings and themes. A coding frame was then developed which captured and encapsulated these themes. As JL’s and NH’s initial analysis made apparent, women’s accounts of the consent procedures used during the pilot were relatively straightforward, with only two key (divergent) perspectives being reported. Staff, however, tended to provide much more in-depth, complex and nuanced perspectives and views. For this reason, coded datasets from the staff interviews were subjected to further, in-depth analysis to identify additional (sub-) themes, together with contextual information needed to aid interpretation of the data. As part of the data analysis process, the qualitative analysis software package NVivo9 (QSR International) was used to facilitate data coding and retrieval.

Ethical approval for the trial and the qualitative research was given by Newcastle and North Tyneside 2 Research Ethics Committee. To safeguard confidentiality, all participants (women and staff) were allocated pseudonyms and these are used below.

## Results

Forty-nine women and 37 staff were invited to participate. Twenty-two (45%) women and 27 (73%) staff were interviewed (10 doctors and 17 midwives). Of the 27 staff interviewed, twelve had been involved in obtaining consent from women for entry into the trial (see Table 2); the remainder (6 clinical midwives, 11 research midwives) were able to make initial approaches about the trial but were unable to obtain consent. Given the focus of this paper is upon soliciting/giving and documenting informed consent, when staff perspectives are reported, we borrow heavily on the accounts of those who had direct responsibility for taking consent from women for trial entry. However, as other staff members did observe consent interactions and had opinions to share, their perspectives, albeit to a lesser extent, have also been included.

**Table 2:** Clinical and Previous Trial experience of staff involved in gaining consent from women into the GOT-IT pilot

Pseudonym	Job title	Previous clinical trial experience (Y/N)	Previous peripartum trial experience (Y/N)
MWA	Research midwife	Y	N
MWC	Research midwife	N	N
Dr A	Obstetric Consultant	Y	N
Dr B	Junior trainee^a^	N	N
Dr C	Junior trainee^a^	Y	N
Dr D	Junior trainee^a^	Y	Y
Dr E	Junior trainee^a^	N	N
Dr F	Senior trainee^b^	N	N
Dr G	Junior trainee^a^	N	N
Dr H	Consultant	Y	N
Dr I	Consultant	Y	Y
Dr J	Junior trainee^a^	Y	N

^a^ < 5 years in training post, ^b^5–7 years in training post

Below, we begin by reporting women’s’ perspectives and views before we go on to consider staff accounts. As we show, while staff, like some women, highlighted potential benefits to using verbal consent at the time of recruitment, those who were responsible for obtaining this consent were reluctant to proceed with enrolment until women had completed and signed a consent form. Analysis of the staff interviews, as we further show, revealed a variety of factors (sub-themes) which helped to account for their unwillingness to forgo written consent at the time of recruitment.

### Women’s experiences and views

All women interviewed described having given written consent at the time of recruitment. Resonating with their broader, and generally positive recruitment experiences [24], most women also presented the completion and signing of the consent form as having been relatively straightforward and unproblematic. Indeed, typically, the giving of written consent was mentioned in passing, and described in a matter of fact tone, as part of a broader account of their recruitment experiences, as Anna’s quote serves to highlight:

“I mean they said, you know, “it’s just going to be a spray and, you know, there is a chance that you’ll get the placebo” and just all of the basic detail. I’m sure if I’d have asked more questions it would have been fine, but I was just happy to have a quick read through and sign it.” (Anna)

However, almost a quarter of the sample (*n* = 5) indicated that they had found the experience of completing and signing the consent form at the time of enrolment burdensome or unwelcome. This included Kate, who had had a 30 h labour which had left her both physically and emotionally exhausted, and who suggested that:

“the only thing personally I would change is signing consent form really… but when you’re in that moment when you have just given birth and you’re in pain, your head’s a bit dazed really to be signing things like that is a bit too much… you have to initial quite a lot [of boxes], you know, to say you’ve understood certain parts and then you have to sign it at the bottom.” (Kate)

A similar, albeit more extreme view, was conveyed by Hannah, who, like Kate, had had a lengthy and exhausting labour, and who intimated that, by asking her to give written consent at the time of recruitment, staff had not been sensitive to her physically compromised state:

“The doctor we’d seen, she was quite hard, she was more interested in me signing the bleeding form while I couldn’t even hold my head up. And the signature on the form, I mean, if you’d seen it, I don’t know, it’s literally a line I just dragged my hand across the thing.” (Hannah)

Others not only described having been too weak and exhausted to complete the consent form with ease, but also highlighted a preference for verbal consent because this would have allowed their entry into the trial to be expedited. This included Trina, who had been induced at 36 weeks due to pre-eclampsia, and who had had a very quick delivery as a consequence, which she described as having resulted in her body having “gone into shock”. She also reported how, at the time of recruitment, she had been extremely worried about her new born baby who had been admitted to the Special Care Baby Unit. Trina presented two overlapping rationales for why she would have benefitted from “giving oral consent with the forms are brought to you at a later stage.” First, like Kate and Hannah, she highlighted her physically compromised state and, hence, her desire to “get on with it”, second, as her quote below makes apparent, she had wanted to be with her baby without delay:

“… obviously I was quite poorly at the time … so I just wanted to get on with it… And I just wanted to get it over with [administration of the spray] so I could be there for my baby.”

### Staff perspectives and views

Mirroring the accounts of those women who had found completion of the consent form at the time of recruitment burdensome, some staff who had witnessed the recruitment and consent process highlighted the importance and value of having the option of using verbal consent procedures. This included MW J who reflected upon how:

“It can be tough, I will say it, because obviously if the lady’s had a long labour and she’s maybe been on the labour ward for, you know, 15, 20 hours or something like that, it must be exhausting. But the good thing is you can get verbal consent so you’re not having to push pieces of paper in front of them… So they can do verbal consent as long as they document it and they’ve explained it all to them and then go back retrospectively and get them to sign after that.” (MW J)

Not only was obtaining verbal consent seen as way of reducing the stress and demands placed on those who, as a result of their birth, had been left exhausted, some staff also highlighted the benefits of expediting the consent process because, as MW N noted “when a woman’s just had a baby there’s a lot going on in their mind and they just want to see their baby.” This view was reinforced by MW D who concluded that, in some situations at least, “I’d much rather get verbal consent and then do the written afterwards” after having observed a woman who, as she described,

“had just wanted to get on with it… but the consent form was quite lengthy. There was a lot on it and a lot of boxes to sign, and she just wanted to crack on because she was in a bit of pain, she’d just had a baby, and the baby was crying.” (MW D)

There was thus, *in principle*, support from some staff who had observed the consent process for offering the option of prospective verbal consent during this perinatal trial. However, amongst those staff who had direct responsibility for obtaining consent from women, there was widespread reluctance, *in practice*, to forgo written consent procedures at the time of trial enrolment. Specifically, of the 12 individuals who had been involved in gaining consent from women, only two indicated a willingness to use a verbal consent when women were enrolled into the trial. Furthermore, during the interview study, which spanned a seven month period, none of those interviewed could point to any instances where verbal consent had actually been used either by themselves or their colleagues. Below we consider reasons for staff reluctance to forgo written consent at the time of enrolment in more detail, beginning with experiential factors (former trial and clinical experiences) before moving onto consider more nuanced ethical issues and concluding with practical considerations.

#### Experiential factors – former trial and clinical experience

“The studies that we’ve worked on have always been, you know, in the antenatal period. And we’ve never had to – with any of our research studies, we give our women 24 hours, and, then, they have time to think about it.” (MW M)

As Table 2 reports, it was not unusual for the staff who took consent during the GOT-IT pilot to have been trial inexperienced or naïve. Furthermore, as MW M (quoted above), like some others, pointed out, even when staff working on GOT-IT had not been trial-naïve, their experiences in trial recruitment and consent had tended to have been restricted to antenatal trials where women are usually given at least 24 h to consider their participation before the informed consent is taken. As various individuals noted, this had meant that, even if staff had had prior experience of obtaining consent from potential participants, this experience had not generally prepared them for GOT-IT. As Dr B and Dr E, like others observed, this was because the circumstances in which recruitment and consent were undertaken were substantively very different to antenatal and other trials, due to recruitment being undertaken in an emergency situation and, relatedly, to there being very limited time available to take informed consent:

“I’ve been involved in quite a few [clinical trials] but not intrapartum, [trials during labour] the majority I’ve been involved with have been antenatal at different stages of pregnancy, which is very different from the [GOT-IT] approach because intrapartum, there’s not the same time for reflection and consideration about whether somebody wants to take part, it’s a little more urgent… there’s this element of someone’s had a baby, the placenta’s not come out, and there’s very limited time to have a discussion about this.” (Dr B)

“it’s been interesting actually being involved in a trial like GOT-IT– well one that is on the delivery unit, in an environment that people aren’t used to having clinical trials. You know, they’re in quite a sort of high stress, quick turnaround, fast paced environment… it’s an interesting environment to consent patients for a clinical trial. Because it’s quite different to a sort of, you know, sit down clinic, have a think about something, then write to me if you’re interested. That’s the thing, it’s kind of a now or never scenario.” (Dr E)

Not only were many individuals new to the kind of “now or never scenario” (Dr E) which the GOT-IT trial had presented, some staff also noted that the majority of those who had been involved in obtaining consent for the trial had been relatively junior and clinically inexperienced. Such a situation, as MW J pointed out, was fairly inevitable because recruitment had, by necessity, to take place as and when a retained placenta was diagnosed, including at night-time and over the weekend, when the more senior and experienced staff were less likely to be on duty:

“I am not saying the doctors [who are obtaining consent for GOT-IT] have never consented anybody to a study, possibly at other hospitals, but in our site certainly, it’s mostly the consultants [who’ve consented into other trials] whereas, for GOT-IT, the registrars, they’ve tended to be ones I’m having to get to do A and B, because they’re the ones on the labour ward, the consultants are at home, they’re not working at the weekends.” (MW J)

Staffs’ relative inexperience of recruiting into peripartum and other trials, as some individuals, including MW F, further reflected, had potentially resulted in a more risk averse approach having been adopted, which had led to a greater onus having been placed on the consent form being completed at the time of recruitment:

“It’s kind of a new thing for the doctors, research, for [site name], is quite new, they’re just getting used to it…So the doctors haven’t recruited per se from [site name] that much… So I think probably more from a point of view that they feel they have to do it, you know, getting the form signed, just to be on the safe-side that they’re doing it correctly.” (MW F)

Indeed, it is notable that one of health professionals (Dr D) who did indicate a willingness to forgo written consent at the time of recruitment had previously been involved in another peripartum trial in an emergency setting [29]. In this trial, as Dr D noted, it was possible to forgo getting written consent at the time of recruitment if the woman was facing a life-threatening postpartum haemorrhage:

“they didn’t have written consent, because that was, well I think it was, as a clinician, if you thought it was in the best interest to give it, I think you just entered the woman into the trial.” (Dr D)

Others indicated that clinical trial research, by its very nature, fostered a risk averse approach: “it’s just the words, clinical trial, I think that make people, don’t know, feel like they need to be extra cautious” (Dr E), because this kind of research entails investigation of drugs or procedures of unknown clinical efficacy in the context of the field of investigation. Indeed, some individuals indicated that, because of this precautionary stance and their prior experiences of having worked on non-perinatal trials where written consent was always obtained prior to administration of the study drug/procedure. As a consequence, soliciting written consent at the time of enrolment had, for them, become a conditioned and taken-for-granted practice:

“I would feel less comfortable about that [taking verbal consent initially and getting written consent later] because you get so used to doing written consents for clinical trials for any sort of significant intervention you get used to doing written consent.” (Dr E)

#### Ethical and perceived legal considerations

As these and other staff noted, reluctance to forgo written consent procedures during GOT-IT often occurred despite their being familiar and comfortable with using verbal approaches when gaining consent from women to go to theatre and undergo other emergency procedures in their routine clinical practice on the labour ward. To help account for these differences in their approaches for obtaining consent, these staff typically drew a strong distinction between trial research and clinical care. In the latter, staff described acting in a patient’s best interests, whereas, in the former, the interests of a wider clinical (i.e. non-trial) population was their paramount consideration:

“Well it is different because – well firstly the fact that the drug that you’re consenting - you’re offering them something which is - you’re not trying to tell them, this is something you should have because it will save your baby kind of scenario. It’s very much, this is something you really don’t have to have, but taking part in the study will potentially give us help in the future to know whether this is a good treatment option or not.” (Dr A)

“I guess it is difficult with all women, I think, isn’t it in labour and delivery and post-delivery about consent, even consenting for things like caesarean sections and forceps, about how much of it can really be truly informed consent, given how tired and things they are…I definitely think there are times when you can question how much the woman is really taking in of what you’re saying. But it’s a bit different when we’re suggesting or saying what we would recommend is definitely what we feel is in their best interests. Which is slightly different to a trial, isn’t it, cause you’re not offering them the best, you know, the gold standard treatment, you’re offering them something slightly different.” (Dr B)

Indeed, as Dr B noted, while there are challenges to obtaining consent from women for emergency procedures in routine clinical practice due to their being exhausted, taking consent in such situations also tends to be seen as being relatively unproblematic and uncontroversial. This is because, as this individual further pointed out, the recommended course of action tends to be delivery of a ‘gold standard’ treatment or procedure of known clinical efficacy. In addition, as Dr F and Dr E indicated below, in many of the emergency situations encountered during their work as obstetricians, there are often no alternative, clinically viable treatment options to offer. Hence, as these individuals intimated, when they gained consent from women in clinical situations, rather than encouraging engaged and reflexive decision-making, they were ostensibly seeking their permission to proceed along a pathway which they saw as being in a woman’s best clinical interests:

“Yeh well that thing of acting in their best interest; I think probably because this issue about not going to theatre is that you could then bleed and maybe need a transfusion, and often if the placenta hasn’t come by about an hour …. you’re then deciding that the course of action is to go to theatre to avoid the woman from bleeding, so you’re deciding, you’re kind of doing a bit of a paternalistic one saying, “well this is what we have to do now, so although I want you to consent to it, I’m not really giving you an alternative”, because we don’t want to leave people’s placenta in do we….if you’re consenting for theatre, you’re thinking well this is in this woman’s best interest to stop her from bleeding, to stop any further complications…so you’re thinking well it’s the best course of action and what else are we going to do.” (Dr F)

“But there are occasions [in emergency situations in routine clinical practice] when it’s extremely rushed and we – you don’t have time to get the patient to sign the consent form. So those you can always justify, cause it’s a matter of life and death to the baby, kind of thing that you would – that it feels okay to miss out on that written consent for the sake of safety.” (Dr E)

Dr F and Dr E were the only two health professionals who indicated or implied that it is acceptable to adopt a paternalistic or directive stance when taking consent from women in routine clinical practice. However others, like Dr F and DR E, were keen to draw a distinction between the kind of consent which they felt was needed to undertake emergency procedures in their everyday work as obstetricians, and what Dr I, below, described as the “higher” (i.e. more informed) level of consent required for a clinical trial such as GOT-IT:

“To be honest, I think a lot of consent taken is, half the signatures on the paper aren’t worth the paper they’re written on. You know, we get consent for caesarean sections in traumatic situations as people are being wheeled down the corridor and everything. But I know the level of consent has to be higher when it’s research, because its research.” (Dr I)

Indeed, such staff were not only keen to emphasise that “the understanding of the woman has to be better because you’re giving a drug or procedure you wouldn’t normally give” as Dr I, quoted above, went on to elaborate, they also noted that, because a women might not gain any clinical benefit from taking part in the trial, she needed to make a more active and voluntary decision to participate:

“I don’t know that I would feel that comfortable [getting verbal consent] about it because it’s not, I suppose that kind of taking verbal consent from people is more when you are acting in their best interests and a trial is a trial and that’s useful for us, but it’s not necessarily in the patients’ best interests. So then it should be that they completely choose it.” (Dr A)

It was partly because staff saw trial participation as requiring active decision-making wherein a woman should “completely choose it” as Dr A put, that some, including Dr I, suggested it was important that the consent form was completed before trial entry. As this individual indicated, this was partly because the act of completing the form helped to create a situation that encouraged a woman to reflect upon the decision she was making:

“I know legally, I think it’s not binding the written consent, but I think it gives them an extra bit of time to firmly say, yes, yes I would like this. So yeah I’d feel a lot more comfortable getting written consent.” (Dr I)

#### Written consent at the point of study entry: protection for staff gaining consent

For others, the importance attached to completing the consent form appeared to be less about creating an environment that fostered informed decision-making, and more about providing tangible “proof” that a women had actively agreed to take part, which could help protect them from subsequent complaints and litigation:

“I don’t feel comfortable with any other consent, I think unless it’s an emergency where, you know, we do sometimes come across emergencies where we have to seek verbal rather than written. I wouldn’t be comfortable seeing verbal consent in this situation [I: and why wouldn’t you] – if she does get side effects from it and she becomes hypotensive or she becomes unwell, or if she starts bleeding, anything. She can blame it on anything. And she might tell me that she hasn’t been informed. And I don’t have any proof of that because there is no documentation. (Dr C)

“So I think, in the occasional patient, that is feeling - like the one saying the other day, ‘am I a guinea pig. I- am I the first person who’s done it?’ I think those people that are- not sceptical, but very inquisitive as to, ‘well why are you doing this to me?’ I dunno, they’d be willing to take part, but I think they would be quick to blame the trial if anything went wrong, even if it was 24 36 hours later. And I think, in those situations I’d feel more comfortable with them having a written consent” (Dr B)

While such staff indicated that they felt more vulnerable to complaints when patients took part in clinical trials, precisely because they might receive a treatment that was not in her best clinical interests, they also suggested that this kind of vulnerability was heightened in a perinatal trial such as GOT-IT. This, as they pointed out, was because recruitment had, by necessity, to be undertaken at a time when a women might be exhausted, distracted and/or under the effects of analgesics. As reported previously [24], despite these mitigating factors, staff conveyed general confidence that they had been able to secure informed consent at the time of recruitment; in large part because of their perceived ability to convey information about the trial in clear and accessible ways [24] and because they saw GOT-IT as a relatively easy trial to understand and explain [24]. However, as Dr A indicated below, some staff also expressed concerns that, because consent was taken at a time when a woman might be physically and emotionally exhausted, she might subsequently forget or struggle to recall all the details of the consent procedure:

“it’s quite an emotional moment – a lot of the time they’re very upset because they’ve had a normal delivery and they’re very pleased with the birth plan and now suddenly they’re being told they may have to go to theatre.. so whatever you tell them at the time, they’re not going to remember, and that’s why it’s important to have the security blanket of something in writing.” (Dr A)

Hence, staff highlighted the need for “the security blanket of something in writing”, as Dr A put it, not only because this evidenced a woman had given her consent at the time of recruitment, but also, as Dr H elaborated, to demonstrate that all of relevant discussions had taken place prior to that which are necessary for making a fully and properly informed decision:

“I mean consent, we have it written down as evidence but actually the consent process is that acknowledgement that the patient does understand what he or she is going forward with and you can never consent without a verbal discussion first… as a practitioner you need to understand yourself that the patient has understood so it needs to be a two way dialogue and writing down is really just a confirmation of that.” (Dr H)

In terms of establishing understanding on the part of the woman, a couple of health professionals also suggested that they attached credence to the completion and signing of the consent form as, in their opinion, the physical act of doing this could be considered a proxy indicator of competence. Indeed, it was precisely because writing was seen as a surrogate for understanding and competence that some health professionals, including Dr H, suggested that they would exclude a women from the trial if she was unable to complete the consent form at the time of recruitment.

“Yeah, no, I think my being uncomfortable isn’t to do with the signing of the paper work in advance. It is to do with the: if they are at a point where they are unable to do that, is that a reflection of their sort of lack of competence actually to make a decision for themselves… I think our concerns as clinicians, well certainly mine would be: if they’re so exhausted that they’re not able to, you know, able to sort of written consent, even you know, just a one signature on one line, em yeah, if they’re not able to do that then I would be concerned that they weren’t actually understanding and weighing up the information, you know, retaining it long enough for them to actually make a decision which was their own sort of competent decision.” (Dr H)

#### Practical reasons for obtaining written consent at the time of recruitment

While staff directly involved in gaining consent from women provided, in the main, ethically (and legally)-informed reasons for wishing to obtain written consent at the time of recruitment, they and their midwifery colleagues had additional, more practical insights and explanations to offer. In particular, staff highlighted the logistical reasons for asking women to complete the consent form at the time of recruitment. This, as they noted, was because, in the delivery wards were most recruitment took place, workloads could be very heavy and unpredictable. Hence, as MW K pointed out, there was a possibility that the staff member responsible for gaining consent from a woman could get called away and might not be able to return a later stage to ask them to complete the form:

“And maybe possibly because they think “well, if this works and we get called away then we need to come back to the room and consent her” whereas they might be called into another room or they might be called into theatre. So it’s maybe just a matter that they’re thinking “well, if I get it done now and the woman’s coherent and she’s able to do it then and there then I’ll just get it done so that it’s, you know, time-specific and I’m not having to return to the room.” (MWK).

Some staff also noted that, if the form needed to be competed retrospectively, there was no guarantee that there would be a staff member on duty at the time who was on the delegation log and, hence, able to take the consent. As MW A further suggested, there was also a possibility that by the time a delegated and trained staff member, such as themselves, had come on duty, a woman might have already been discharged:

“if it happens on a Friday and you’re not in till the Monday and the woman’s gone home, it’s a bit more of a nuisance.” (MW A)

## Discussion

This study has explored patient and staff experiences of, and views about, a consent process which has been advocated for use in some trials involving recruitment in emergency situations: the option of using initial verbal consent followed later by written consent [21–23, 30]. As we have shown, women who took part in the GOT-IT pilot endorsed use of initial verbal consent, especially those who had been left exhausted by their births and/or who had wanted to truncate the consent process in order minimize time spent away from their new born. In keeping with these women’s accounts, some staff who had observed practices of gaining consent during the pilot also highlighted the potential benefits of using a verbal approach at the time of enrolment. However, discordant views were expressed by the vast majority of staff who had direct responsibility for enrolling women into the trial, despite these individuals wanting to expedite recruitment and reduce the burden placed on potential recruits [24]. To account for their resistance to forgo prospective written consent, these staff members drew a distinction between research and clinical care, one which is well recognised in bioethics literatures [31, 32]. In doing so, staff emphasised the need to engage women in a reflexive decision-making process to help ensure they made an active and voluntary choice to take part in a trial that, by its very nature, could not guarantee that administration of the trial drug would enable the placenta to be delivered. Staff also highlighted the role that completing and signing the consent form at the time of enrolment could play in helping to reinforce and provide evidence of a reflexive and informed decision-making process. While most staff cited their ethical responsibilities to women as the rationale for asking for prospective written consent, their accounts also revealed a seemingly risk averse approach. Specifically, some staff highlighted a (mis)perception that the absence of a signed consent form at the time of trial enrolment could expose them to greater risk of litigation were a woman to experience a problem or complication during the trial. Others voiced concerns that it might prove difficult to get the form completed after the trial, especially if a woman had already been discharged.

In keeping with these findings, other research also suggests that concerns about litigation may impact on the conduct and preferences of staff involved in obtaining consent for participation in emergency trials. This includes survey research undertaken with Polish emergency stroke clinicians which found that, despite there being no legal requirement to do so, more than 50% sought parallel consent from patients’ relatives [4]. Moreover, while most clinicians endorsed use of a truncated consent process, only 7.5% supported forgoing prospective written consent [4]. While highlighting potentially important issues, the authors, however, were unable to provide explanations for these findings. In another questionnaire study that explored health professionals’ views about using deferred consent in paediatric and neonatal emergency care trials, Woolfall and colleagues found that staff held negative perceptions and views about this consent pathway. Interestingly, however, these negative perceptions were restricted to staff who had no prior experience of deferred consent; practitioners experienced in this method were much more positive about its use [19]. Indeed, companion qualitative research highlighted how health professional views about using deferred consent could change in light of their experiences of using this method and seeing a positive impact on recruitment, decision-making capacity, and practitioner-parent relationships [18]. While Woolfall et al.’s research focused on deferred consent procedures, it is relevant to the current study as it draws attention to how staff (in)experience may impact on their confidence and willingness to recruit into trials without obtaining prospective written consent. Indeed, in keeping with Woolfall et al.’s observations, some of the individuals we interviewed suggested that reluctance to forgo prospective written consent during the GOT-IT pilot had arisen in part from the relatively junior status and inexperience of most staff who had been involved in obtaining consent from women for trial participation. Further paralleling Woolfall et al.’s observations, it is interesting to observe that one of the staff who said they were willing to forgo verbal consent for the GOT-IT trial had had previous experience with another peripartum trial in an emergency setting which used prospective verbal consent.

Staff (in)experience is an important issue to consider in the design and delivery of future trials which require truncated recruitment and consent approaches to be used, particularly where the responsibility to consent may fall to junior clinical staff who are emergency trial naïve. This is particularly relevant to peripartum trials where the junior clinical trainees are often based in delivery suites and responsible for dealing with peripartum emergencies. Because of time-critical nature of recruitment in these kinds of trials where delaying definitive management risks patient safety, junior staff may thus best placed to obtain consent. As the findings of this and other studies indicate, staff who are inexperienced in using alternatives to prospective written consent may benefit from training and support to increase their confidence and willingness to use alternative consent approaches. This training and support could focus on raising staff awareness and understanding of ethical review processes and of how, and why, they are legally protected when alternatives to prospective written consent are used [17]. During training, staff may also be encouraged to consider how obtaining prospective written consent may lead to avoidable delays to starting treatment, which as Roberts and colleagues argue, may obscure real treatment benefit as well as resulting in avoidable morbidity and mortality is some kinds of emergency trials [9]. Given the relatively high turnover of junior clinical staff in obstetrics and other clinical specialities, we would recommend that training and support continues throughout a trial’s recruitment phase. We would also recommended that, in each trial site, as many staff as possible are trained up to take consent. As our findings indicate, this might help alleviate pressures staff feel to obtain prospective written consent due to their concerns that there might not be suitably delegated and trained staff available to obtain this consent retrospectively.

Commentators advocating use of initial verbal consent in emergency medical research have also recommended that this consent be observed and documented by a patient advocate [21, 22], a role filled by health care professional who is independent of the research team, and who is in a position to interpret the patient’s condition and responses to the researcher [22]. Such individuals were not used in the GOT-IT pilot, and it is possible that, if they had, this might have helped alleviate staffs’ perceived need to get written consent at the time of trial enrolment. In particular, having an independent witness observe the consent process might have helped address staff concerns about needing to evidence that all relevant information had been given and that all relevant discussions had taken place. In addition, health professionals might not have attached such credence to the signing and completion of the consent form, if competency could have been confirmed by an independent witness. Use of patient advocates could be considered in future trials involving recruitment in emergency situations, as well as being the subject of future (qualitative) research. However, in peripartum trials, such as GOT-IT, logistical issues may also need to be considered and addressed. This includes the potential difficulties of accessing an independent health care professional, especially if recruitment needs to be undertaken at times (e.g. late at night) when staff availability may be limited and where delaying recruitment to the trial potentially risks patient safety.

By using an open-ended, qualitative approach, it has been possible to provide a level and depth of insight that has not been possible in studies that have solicited staff views using questionnaire designs [4, 19]. An additional study strength is our decision to draw upon the perspectives and experiences of women as well as trial staff. By showing that women and staff may hold discordant views, our study supports recommendations, made previously [24] and by others [15, 17–19], that different stakeholder groups, including patients and healthcare providers, should be involved in the development and evaluation of study protocols and consent procedures used in future emergency trials. Of the 27 staff we interviewed, only twelve were able to consent women into the trial and, given the focus of this paper, these staff members’ accounts were drawn upon most heavily in our analysis. Because this is a relatively small sample size, we cannot guarantee data saturation was achieved; however, we have been able to capture some of the complexities and nuances informing staff decision-making and consent preferences at the time of trial enrolment. It should be taken into account, however, that some staff who obtained consent might have used their interviews to present themselves in a favourable light; this includes the possibility that they provided post-hoc rationalizations to justify their decisions to press for written consent at trial enrolment.

## Conclusion

Alternative consent pathways, such as use of initial verbal consent, have an important role to play in advancing emergency medicine research. However, while potential trial recruits may appreciate use of such approaches, they may present unintended logistical challenges and ethical and legal concerns to recruiting staff. Our research highlights the importance of drawing upon a stakeholder perspective at an early stage in the research process to understand and explore how alterative consent pathways are used. Furthermore, our study highlights the potential value of using the insights of those with practical experience of alternative consent pathways to develop and offer staff appropriate training and support.

When considering the generalizability and broader applicability of our findings, it should also be taken into consideration that our research cohered around one trial. It is possible that some of the issues and concerns highlighted by the staff we interviewed might be heightened in, or restricted to, peripartum trials, such as GOT-IT because obstetrics is one of the most litigious specialities. Staffs’ concerns about litigation as a result of adverse events occurring during normal clinical practice might therefore have influenced their views about gaining consent from women to a clinical trial where the perceived risk and benefits of trial participation are less clear. Furthermore, many women are given a potent analgesia during labour and staff may have been uncertain whether this might have affected their ability to understand the trial and implications for potential participation. In addition, as reported previously [25], GOT-IT involved investigation of what staff considered a familiar, relatively low risk and benign drug. Consequently, recruitment to the trial was regarded as having been relatively unproblematic and uncontroversial. We would therefore recommend that further research be undertaken as part of the other trials offering verbal consent at the time of enrolment, including those involving “more risky” or unfamiliar interventions. The potential value of undertaking further research as part of peripartum trials delivered in other countries, where concerns about patient autonomy and litigation may be different, could also be considered.

## Abbreviations

GOT-IT: Glyceryl Nitrate for Retained Placenta
GTN: Glyceryl trinitrate
RCT: Randomised Controlled Trial
